# The Study of Cognitive Function and Related Factors in Patients With Heart Failure

**DOI:** 10.5812/nms.12442

**Published:** 2013-09-15

**Authors:** Atefeh Ghanbari, Fatemeh Moaddab, Arsalan Salari, Ehsan Kazemnezhad Leyli, Mitra Sedghi Sabet, Ezzat Paryad

**Affiliations:** 1 Social Determinants of Health Research Center, Guilan University of Medical Sciences, Rasht, IR Iran; 2 Cardiology Department, Guilan University of Medical Sciences, Rasht, IR Iran; 3 Biostatistics Department, Social Determinants of Health Research Center, Guilan University of Medical Sciences, Rasht, IR Iran; 4 Nursing Department, Nursing and Midwifery School, Guilan University of Medical Sciences, Rasht, IR Iran

**Keywords:** Executive function, Cognitive disorders, Heart failure

## Abstract

**Background:**

Cognitive impairment is increasingly recognized as a common adverse consequence of heart failure. Both Heart failure and cognitive impairment are associated with frequent hospitalization and increased mortality, particularly when they occur simultaneously.

**Objectives:**

To determine cognitive function and related factors in patients with heart failure.

**Materials and Methods:**

In this descriptive cross-sectional study, we assessed 239 patients with heart failure. Data were collected by Mini Mental status Examination, Charlson comorbidity index and NYHA classification system. Data were analyzed using descriptive statistics, Kolmogorov-Smirnov test, chi-square test, t-test and logistic regression analysis.

**Results:**

The mean score of cognitive function was 21.68 ± 4.51. In total, 155 patients (64.9%) had cognitive impairment. Significant associations were found between the status of cognitive impairment and gender (P < 0.002), education level (P < 0.000), living location (P < 0.000), marital status (P < 0.03), living arrangement (P < 0.001 ), employment status (P < 0.000), income (P < 0.02), being the head of family (P < 0.03), the family size (P < 0.02), having a supplemental insurance (P < 0.003) and the patient’s comorbidities (P < 0.02). However, in logistic regression analysis, only education and supplementary insurance could predict cognitive status which indicates that patients with supplementary insurance and higher education levels were more likely to maintain optimal cognitive function.

**Conclusions:**

More than a half of the subjects had cognitive impairment. As the level of patients cognitive functioning affects their behaviors and daily living activities, it is recommended that patients with heart failure should be assessed for their cognitive functioning.

## 1. Background

Heart failure (HF) is a clinical syndrome in which the heart cannot pump enough oxygenated blood to meet the body’s metabolic needs (1). The incidence of HF was tripled in the past 2-3 decades (2, 3) and now millions of people are involved around the world (3). It is estimated that about one percent of people over fifty years and 10% of population over eighty years have HF (4). Also, about 2% of the health care budgets are spending for patients with HF in developed countries (5).

Heart failure is associated with frequent hospital admissions, decreased quality of life, significant morbidity, and increased mortality (6). In addition to dyspnea, fatigue and depression; cognitive impairment is prevalent among patients with HF and negatively affects their quality of life and self-care capabilities (7). This problem hampers the patient’s understanding and interpretation, hinders learning abilities, decreases the patients reaction time and decision-making in daily living activities (8).

Cognitive impairment usually accompanies heart failure (6) and both are associated with frequent hospital readmissions and increased mortality, especially when they occur simultaneously (9). Because 80% of patients with heart failure are older than 65 years, and cognitive disorders are common in these ages, the HF induced cognitive impairment is usually underestimated by physicians (10).

Although several investigations exist about the prevalence, risk factors and impacts of cognitive impairment in patient with HF, the results are inconsistent about the association between HF and the degree of cognitive dysfunction (7, 9, 11, 12). A review of the literature on the cognitive functioning and chronic heart failure has reported that approximately 20-50% of patients with HF have shown the symptoms of cognitive dysfunction (11). However, some of studies have reported that patients with heart failure may be normal regarding cognitive functions. In a study performed by Cameron et al. 25% of patients with HF had a normal cognitive functioning (13). Others are yet in doubt whether these cognitive problems are associated with heart failure or with aging itself (7, 14).

Despite the increasing number of patients with HF and various degrees of cognitive dysfunction in these patients, factors associated with impaired cognitive functioning are controversial. Pressler et al. in a study of the factors affecting cognitive deficits in chronic HF have reported that age, gender and severity of HF were significantly associated with cognitive functioning (7). In another study, Bennett and Sauve have reviewed cognitive deficits in patients with HF and reported that age and ejection fraction can predict the level of cognitive functioning (15). Other studies have also reported that age, severity of hypertension and heart failure (6), the severity of depressive symptoms and the levels of hemoglobin (12), are associated with cognitive function in patients with HF. It seems that different psychosomatic and socio-cultural factors affect the severity of cognitive impairment in patients with HF.

## 2. Objectives

This study aimed to evaluate the level of cognitive functioning and it influencing factors in patients with heart failure.

## 3. Materials and Methods

This cross-sectional study was conducted on patients referred to Dr. Heshmat hospital in Rasht, Iran, from mid-September to the end of December 2012. Sample size was calculated based on a previous study in which the correlation between cognitive impairment and HF was reported to be 0.32 (13). Then 248 samples were estimated to be needed based on the following parameters (α = 0.05, β = 0.90 and r = 0.32). Finally, the data of 239 subjects was analyzed because nine questionnaires were responded incompletely. The needed samples were selected consecutively from the patients referred to the cardiac care ward and specialty clinic of the before mentioned hospital.

All patients with a medical diagnosis of HF confirmed by a specialist, evidence of cardiac dysfunction on echocardiography report, an ejection fraction of 25% or higher, HF class I, II or III based on the New York Heart Association (NYHA) classification, age 45 years and over, ability to speak and read and write in Persian language and consent for participating were entered the study. Also, exclusion criteria were a history of neurological disorder (i.e. cerebrovascular accident, transient ischemic attack, dementia or impaired short-term memory), emotional instability, hearing and visual disorders, a history of working in hospitals or medical centers, a history of formal educations on treatment and self-care in HF.

The instrument for data collection included three parts. The first part included questions on demographic data including age, gender, marital status, education level, employment status, housing, living arrangement, family size, average monthly income, and insurance status (including having a supplemental insurance). Also there were questions related to the disease including duration of illness, history of education about the disease and self-care needs, number of hospitalizations, the severity of heart failure based on the NYHA classification system (16-18), the ejection fraction, having a diagnosis of valvular heart disease, ventricular abnormalities, levels of hemoglobin and blood pressure and other accompanied disorders. The second part included the Charlson’s Comorbidity Index and the third part included a tool for Mini Mental Status Examination (MMSE) to assess the patients’ cognitive function.

The Charlson’s Comorbidity Index was designed by Charlson in 1987 and based on the ninth edition of international classification of diseases (ICD-9). The index includes 19 disease conditions and specifies a score from one to six to the disease based on its potential mortality. This score then is ranked in four categories including zero, 1-2, 3-4, and five and more. A higher score indicates a greater number of accompanied disorders and comorbidities (19).

The MMSE Scale was first designed in 1975 by Folstein et al. (20) and included 20 questions with correct or incorrect answer. This scale assesses the short-term memory, awareness, attention, calculation, motor and language skills. Each correct answer gives a score of one and the maximum score is score less than 22 is indicative for cognitive impairment (21). The Persian version of this tool has been validated by Foroughan et al. (20) and Seyedian et al. (21). All data were collected by the second researcher through interviews with patients and reviewing the patients’ medical records.

### 3.1. Ethical Considerations

This study was approved by the Ethics Committee of Research Deputy in Guilan University of Medical Sciences. Written informed consent was obtained from all of the participants at the beginning of the study. All pparticipants were informed of voluntary nature of participation and were assured about confidentiality of their personal information.

### 3.2. Data Analysis

Data analysis was conducted using the SPSS statistical software version 13. Descriptive statistics (frequency, percentage, mean and standard deviation) and inferential statistics including Kolmogorov-Smirnov test was used for testing the normality of data distribution. Independent sample t-test was used to compare cognitive status score with socio -demographic characteristics and diseases related variables. Chi-square was used for variables with non-normal distribution. Also the logistic regression with enter method was used to determine the predictors of cognitive functioning. A p-value of less than 0.05 was considered significant in all testes.

## 4. Results

Demographic characteristics of the participants with and without cognitive impairment were summarized in Table 1. The mean age of the patients was 59.04 ± 9.91 years. The mean age of the patients with and without cognitive impairment was 61.37 ± 9.89 and 54.75 ± 8.44 years respectively (P = 0.16). The mean and standard deviation of cognitive function score was 21.68 ± 4.51. 

The mean duration of illness in participants with cognitive impairment was 4.19 ± 5.95 years and in those without cognitive impairment was 3.43 ± 5.56 years (P = 0.69). From the total sample, 155 patients (64.9%) had cognitive impairment and 84 (35.1%) had no cognitive impairment. Significant associations were found between the status of cognitive impairment and demographic variables such as gender (P < 0.002), education level (P < 0.000), living location (P < 0.000), marital status (P < 0.03), living arrangement (P < 0.001 ), employment status (P < 0.000), income (P < 0.02), being the head of family (P < 0.03), the family size (P < 0.02), having a supplemental insurance (P < 0.003) and the patient's comorbidities (P < 0.02).

Also, the frequency of cognitive impairment was higher among patients with higher levels of heart failure. So that 60% of patients with class I heart failure and 72.4% of patients with class III HF had cognitive impairment. However, no significant association was found between the severity of HF and cognitive impairment (Table 1). 

**Table 1.** Correlation between Demographic Variables and Cognitive Function

Variables	Having Cognitive Impairment		P value
	Yes, N, (%)	No, N (%)		
**Gender**			0.002^a^
Female	59 (78.7)	16 (21.3)	
Male	96 (58.5)	68 (41.5)
**Marital status**			0.034^a^
Married	134 (62.6)	80 (37.4)	
Single (including widowed and divorced)	21 (84)	4 (16)
**Education level**			0.001^a^
Can read and write	144 (86.7)	22 (13.3)	
Under diploma	7 (41.2)	10 (58.8)
Diploma and higher	4 (7.1)	52 (92.9)
**Employment status**			0.000^a^
jobless	9 (90)	1 (10)	
Household	55 (80.9)	13 (19.1)
Non official	53 (63.9)	30 (36.1)
Worker	14 (73.7)	5 (26.3)
Clerk	1 (7.1)	13 (92.9)
Retired	23 (51.1)	22 (48.9)
**Living arrangement**			0.001^b^
Live alone	10 (83.3)	2 (16.7)	
With spouse	45 (75)	15 (25)
With children	11 (100)	0
With spouse and children	88 (57.5)	65 (42.5)
With parents	1 (33.3)	2 (66.7)
**Living location**			0.001^a^
Urban	88 (56.8)	67 (43.2)	
Rural	67 (79.8)	17 (20.2)
**Income, Mean ± SD**	13.16 (2.81)	5.69 (4.52)	0.017^c^
**Family size, Mean ± SD**	3.46 (1.65)	3.55 (1.29)	0.016^c^
**Being the head of family**			0.034^a^
Yes	110 (61.1)	70 (38.9)	
No	45 (76.3)	14 (23.7)
**Having a complementary insurance**			0.003^a^
Yes	51 (53.7)	44 (46.3)	
No	104 (72.2)	40 (27.8)
**Severity of heart failure**			0.18^a^
Class I	57 (60)	38 (40)	
Class II	35 (61.4)	22 (38.6)
Class III	63 (72.4)	24 (27.6)
**Number of accompanied disorders**			0.023^a^
0	69 (65.7)	36 (35.3)	
1-2	28 (50)	28 (50)
3-4	46 (71.9)	18 (28.1)
Five and more	12 (85.7)	2 (14.3)

^a^ Chi-square

^b^ Fisher’s exact test, c t-test

^c^ t-test

All the demographic variables were entered the model of regression analysis but only the patient’s education level and having a supplemental insurance could significantly predict cognitive functioning. So that patients with higher education levels had a higher chance of having a normal cognitive function than ones with education level under the diploma and patients with ability to write and read (OR = 0.01, CI = 0.002 - 0.04, and OR = 0.09, CI = 0.02 - 0.46 respectively). Also, patients with a supplemental insurance had a higher chance to have a normal cognitive function than patients without a supplemental insurance (OR = 3.87, CI = 1.4 - 10.7) (Table 2). 

**Table 2.** Regression Coefficients of Predictors of Cognitive Function in the Subjects

Predictors	β	SE	Odds Ratio	95% Confidence Interval of OR	P Value
**Education level**					
Ability to read and write	-4.74	0.77	0.01	0.002, 0.04	0.001
Under diploma	-2.4	0.83	0.09	0.02, 0.46	0.004
Diploma and higher	Reference	-	-	-	-
**Having a complementary insurance**					
Yes	1.35	0.52	3.87	1.4, 10.7	0.009
No	Reference	-	-	-	-

## 5. Discussions

The present study showed that most patients with HF had cognitive impairment. This finding is consistent with several previous studies. These studies have reported that patients with chronic HF had more problems in memory, speed of psychomotor reactions and functional performances compared to healthy participants. They have also reported that more severe heart failure was associated with greater degrees of cognitive impairment (6, 7, 9, 11, 14, 22). Although the mechanisms of cognitive dysfunction is not fully understand; however, a decrease in cerebral perfusion secondary to the decrease in cardiac output, may explain this phenomenon (7, 14).

In the present study, the prevalence of cognitive impairment was higher among female patients, those with lower levels of literacy, patients living in rural areas, singles, and housekeepers, those living with their children, patients without a supplementary insurance, and those who were not the head of the family. Such findings were inconsistent with the study of Pressler et al. in which cognitive impairment was more prevalent in male patients and those with higher ages (7). Some studies have also reported that only the age of patients with chronic HF is directly correlated with cognitive impairment (6, 10, 15). It seems that some of individual or social factors affect the patients’ independence, personal abilities, and confidence that consequently affect the patient’s cognitive function. On the other hand, it seems that women, patients in rural areas and those with lower education usually are not adequately engaged in their own caring and therapeutic programs because of their lower levels of knowledge and unawareness. 

In the present study, the number of accompanied disorders (based on Charlson’s Comorbidity Index of five and more) showed a significant association with cognitive status. This finding was inconsistent with the results of Pressler et al. in which no association was observed between comorbidities and cognitive status (7). It seems that the comorbidities affect the patients’ psychological functioning and decision-making ability. Then more comorbidities would result in a weaker cognitive function. 

The present study showed that the prevalence of cognitive impairment has increased with increasing the severity of HF. However, the severity of HF was not known as a predictor of HF. This finding is not consistent with some of the previous studies that concluded that the severity of HF is significantly related to the severity of cognitive impairment (6, 7, 14, 15).

The results of the regression analysis showed that education level and having a supplemental insurance were the significant predictors for cognitive functioning in patients with HF. Such finding was not consistent with previous studies. A study by Pressler et al. reported that age, gender and the severity of HF are the predictors of cognitive functioning in heart failure (7). Another study has also predicted that depression and the level of the serum hemoglobin can predict cognitive impairment in patients with HF (12). 

In conclusion, more than a half of the subjects in the present study had cognitive impairment. As the level of the patients cognitive functioning affect their behaviors and daily living activities, it is recommended that patients with heart failure should be assessed for their cognitive functioning. The present study revealed that education level and the patients’ type of insurance as predictors for cognitive functioning which consequently may affect their level of participation in implementing caring plans. Therefore, the authorities should try to provide supportive facilities such as their levels of insurance to decrease the patients stress and to improve their cognitive functioning. Also the health professionals should be trained about the effects of heart failure on the patients’ cognitive performance and its consequences.

The instrument used to assess cognitive impairment and other confounding variables such as physical deficits may be affected on the results of the present study. A more precise instrument may lead to a better diagnosis. Then, researchers should focus to design special tools for evaluating the cognitive function in patients with HF.
